# Supplementary material for: XAANTAL1 Reveals an Additional Level of Flowering Regulation in the Shoot Apical Meristem in Response to Light and Increased Temperature in Arabidopsis
Source: Int J Mol Sci. 2023 Aug 14;24(16):12773. doi: 10.3390/ijms241612773 (PMC10454237; doi:10.3390/ijms241612773)
Supplement: Supplementary file 1 [file ijms-24-12773-s001.zip › ijms-2537731-supplementary.pdf]

A

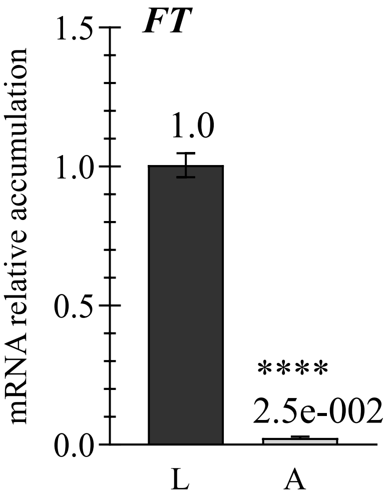

B

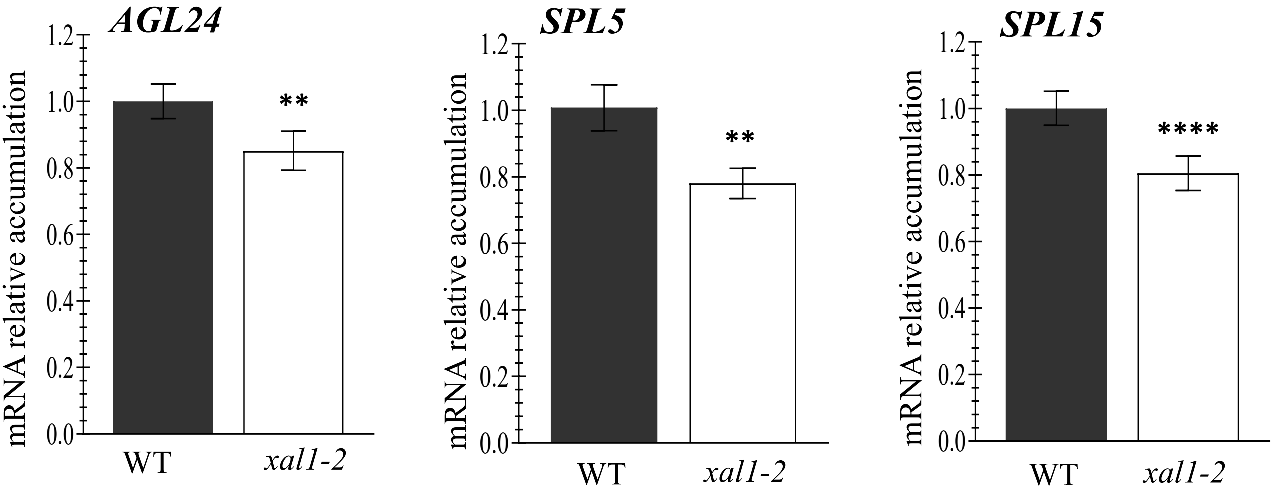

Figure S1. Expression analysis of additional flowering genes. (**A**) *FT* is highly accumulated in leaves (L) compared to apices (A) collected from WT control plants. (**B**) XAL1 positively regulates *AGL24, SPL5,* and *SPL15.* RT-qPCRs were performed with RNA extracted from shoot apices of WT and *xal1* plants. *PDF2* and *RNAH* were used as qPCR internal controls. Data represent the mean value ± standard error of three biological replicates. Statistical analysis was performed by a T-Student Test (** $p<0.01$; ****$p<0.0001$). 14-day-old plants were used in all cases.

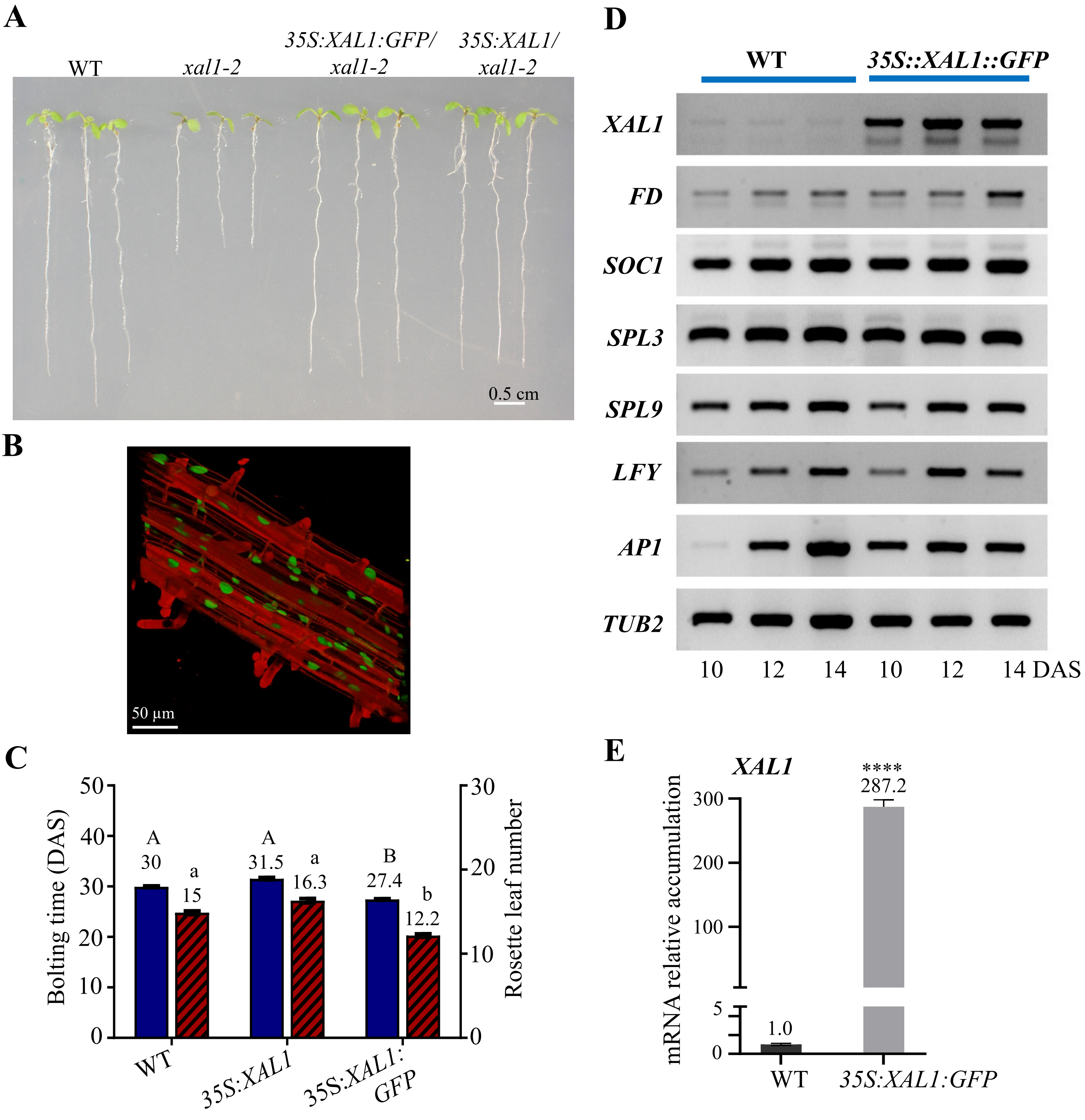

Figure S2. Expression analysis and effect of the *XAL1* overexpression lines. (**A**) Complementation of the *xal1-2* root phenotype with *35S::XAL1::GFP* and *35S::XAL1,* respectively. (**B**) *XAL1* expression in the nuclei of *35S::XAL1::GFP/xal1-2* roots observed by confocal microscopy. Z-stack image of 165 optical sections, step-size of 0.8 µm. (**C**) Flowering time of both overexpression lines in the *xal1-2* background compared to WT, under LD photoperiod. Statistical analysis was performed by a Kruskal-Wallis test followed by a Dunn's analysis $P < 0.001$ (n = 39-46). (**D**) Expression analysis of some flowering time genes in the complete aerial section of the *35S::XAL1::GFP/xal1-2* seedlings compared to the WT. Plants were grown on MS plates for 10, 12, and 14 days after sowing (DAS). *TUB2* was used as an internal RT-PCR control. (**E**) Relative *XAL1* accumulation levels in the aerial section of the *35S::XAL1::GFP /xal1-2* line compared to WT seedlings of 14 DAS. *RNAH* and *UPL7* genes were used as internal controls. Statistical difference was obtained by a T-student test ****$P < 0.0001$ (n = 3 biological replicates).

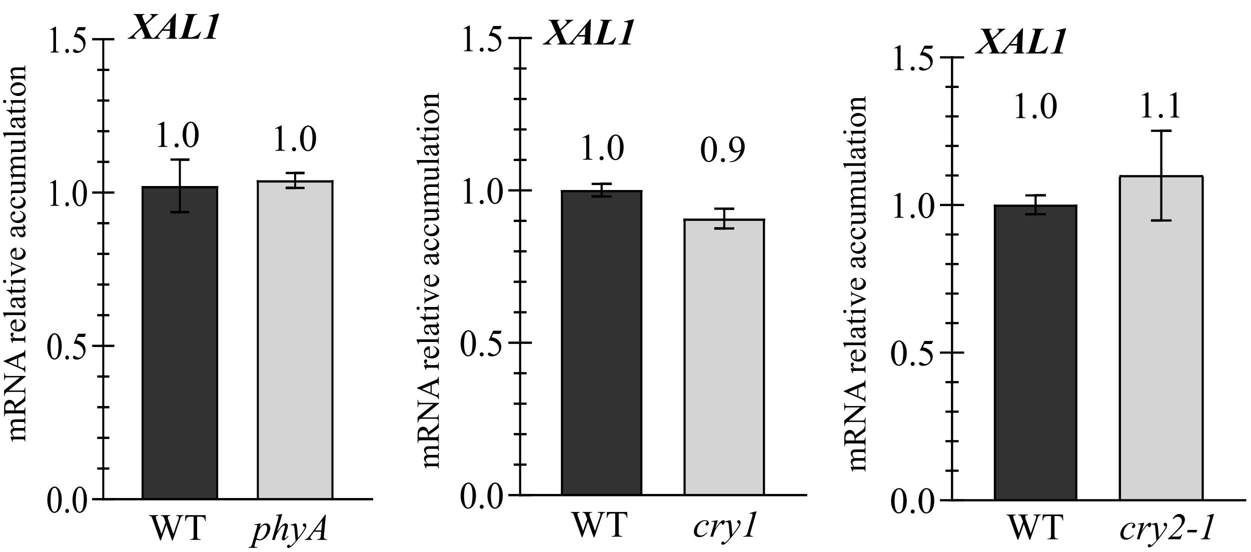

Figure S3. PHYA, CRY1 and CRY2 do not regulate *XAL1* in apices from 18 DAS plants grown under LD photoperiod. RT-qPCR were performed using three replicates in each case. *PDF2* and *UPL7* were used as internal constitutive controls. Data represent the mean value ± standard error. Statistical analysis performed with a T-student test.

A

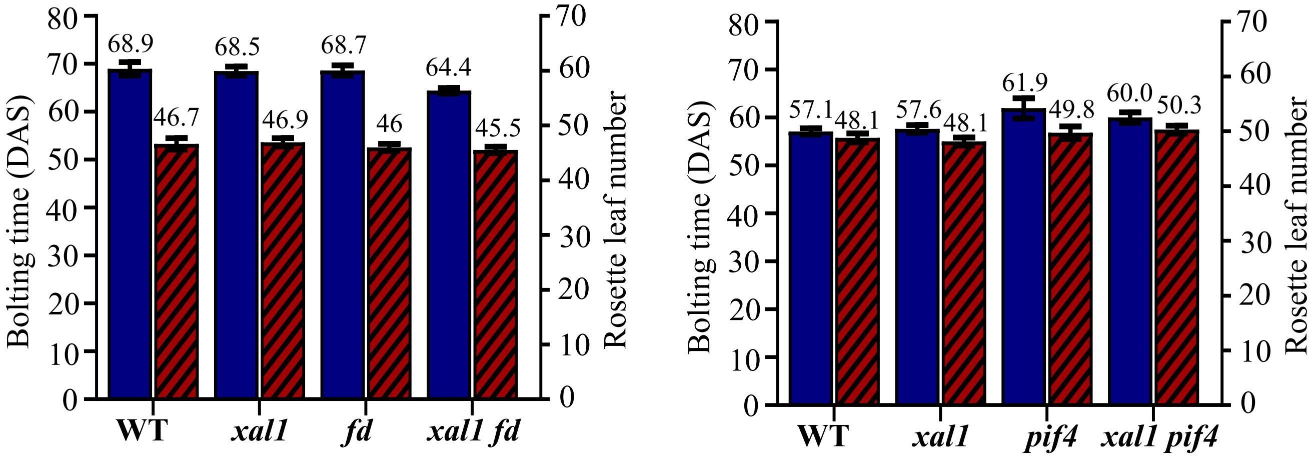

B

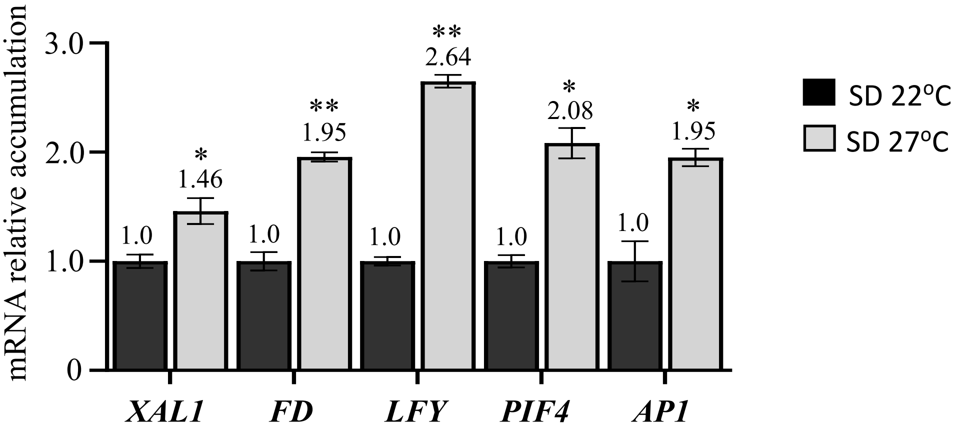

Figure S4. Control experiments of flowering time under SD at 22 ºC and induction of key flowering genes at 27 ºC. **(A)** Single and double mutants show no significant difference between them and the WT when grown under SD photoperiod at 22 ºC. Blue bars correspond to the bolting time, and stripped red columns show the rosette leaf number (left graphic n = 30-35, right graphic n = 16-34). **(B)** RT-qPCR of some flowering genes in WT shoot apices from plants grown under SD at 22 or 27 ºC, respectively. Statistical differences were obtained by a T-student test *P < 0.05; **P < 0.01. *RNAH* and *UPL7* genes were used as internal qPCR controls. Data represent the mean value ± s. e.

Table S1. Primers used for genotyping, semiquantitative RT-PCR and RT-qPCR

Genotyping	Sequence (5'-3')	Reference
co-1<br>*CO-F*<br>*CO-GT1*	 CCATTGTCGTTGTAGTGAGT<br>CTGCAAACCCACTTGCTAGA	 (Han et al., 2008)
fd-3<br>*Salk_LBb1*<br>*FD-F*<br>*FD-R*	 GCGTGGACCGCTTGCTGCAACT<br>AGCCAGCGCCGGAATTCAAG<br>CTCTCTGCGTGTAGGATACTAC	
phyB<br>*Salk_LBb1*<br>*PHYB-F*<br>*PHYB-R*	 GCGTGGACCGCTTGCTGCAACT<br>GCAATGGGAAGTTTGTTCAGG<br>TGACTTACAATCGCGTTTATGAC	
pif4<br>*Sail_LB3*<br>*PIF4-F*<br>*PIF4-R* (EMO207)	 TAGCATCTGAATTTCATAACCAATCTCGATA<br>CAC<br>ACCAACGATCAGGATCAAACC<br>TCCAAACGAGAACCGTCGGT	(Leivar et al.,<br>2008)
soc1<br>*Salk_LBb1*<br>S-138131-F<br>S-138131-R	 GCGTGGACCGCTTGCTGCAACT<br>TACACAAACCCTTTATCCTCG<br>AGCACTGAGAGGTCAAAGGC	
xal1-2<br>GK T-DNA<br>NASC12-LP<br>NASC12-RP	 CCCATTTGGACGTGAATGTAGACAC<br>ACCCAAACGTCAAATCATCAG<br>CTTCATTCCGAAACACAATGC	Tapia-López et al.,<br>2008
**RT-PCR**		
XAL1<br>XAL1-F<br>XAL1-R	 ATGGCTCGTGGAAAGATTCAGC<br>GAACTGAAATATTTCACTTGGCA	
FD<br>FD-F<br>FD-R	 CTCTCTGCGTGTAGGATACTAC<br>AGCCAGCGCCGGAATTCAAGC	
SOC1<br>SOC-F<br>SOC-R	 TGAGGCATACTAAGGATCGAGTCAG<br>GCGTCTCTACTTCAGAACTTGGGC	
LFY<br>LFY-F<br>LFY-R	 AGCATCCGTTTATCGTAACGG<br>ACGATCCGGTACAGCTAATAC	
AP1<br>AP1-F<br>AP1-R	 GCACCTGAGTCCGACGTC<br>CGGCGAAGCAGCCAAGG	
SPL3		

SPL3-F<br>SPL3-R	TCATGTTCGGATCTCTGGTC<br>ACCATGTCGTAGGTTTAGCAG	
SPL9<br>SPL9-F<br>SPL9-R	 GATCTACTTCGAGGACGGTG<br>CACCATTTTCGTAAAGCGAAG	
TUB2<br>Tub-F<br>Tub-R	 AGGACTCTCAAACTCACTACC<br>TCACCTTCTTCATCCGCAGTT	
**qPCR**		
AGL24-F<br>*AGL24-R*	GAGGCTTTGGAGACAGAGTCGGTGA<br>AGATGGAAGCCCAAGCTTCAGGGAA	(Liu et al., 2008)
AP1-F<br>*AP1-R*	CATGGGTGGTCTGTATCAAGAAGAT<br>CATGCGGCGAAGCAGCCAAGGTT	(Liu et al., 2008)
FD-F<br>*FD-R*	CTCAAGAGACAACAAGATCAG<br>CACTTCTTCATGAGACAATCTC	
FT-F<br>*FT-R*	GGTGGAGAAGACCTCAGGAA<br>ATATTCTCGGAGGTGAGGGT	
FUL-F<br>*FUL-R*	GTTCTTCTGCCTCAATACTG<br>GAGATAGTTCTACTCGTTCGT	
LFY-F<br>*LFY-R*	ATCGCTTGTCGTCATGGCTG<br>GCAACCGCATTGTTCCGCTC	(Han et al., 2008)
PDF2-F<br>*PDF2-R*	TAACGTGGCCAAAATGATGC<br>GTTCTCCACAACCGCTTGGT	(Czechowski et al., 2005)
PHYB-F<br>*PHYB-R*	ATGCACCATCTCAAGAGTGG<br>GCCATTCTGAATTCTGTGCG	
RNAH-F<br>*RNAH-R*	CCATTCTACTTTTTGGGCGGCT<br>TCAATGGTAACTGATCCACTCTGATG	(Czechowski et al., 2005)
SOC1-F<br>*SOC1-R*	AGCTGCAGAAAACGAGAAGCTCTCG<br>GGGCTACTCTCTTCATCACCTCTTCC	(Liu et al., 2008)
SPL3-F<br>*SPL3-R*	TCATGTTCGGATCTCTGGTC<br>TTCCGCCTTCTCTCGTTGTG	
SPL4-F<br>*SPL4-R*	CAGTAGGTTTCATGACCTCC<br>CCATAAGTACTCTCACCAGAG	
SPL5-F<br>*SPL5-R*	GCCAAGCAGTATTACCGCAG<br>TCTGGTAGCTCATGAAACCTG	
SPL9-F<br>*SPL9-R*	GGAATTTGACCTAGAGAAAAG<br>CACCATTTTCGTAAAGCGAAG	
SPL15-F<br>*SPL15-R*	CAGCCACCGCCCATTTCAAC<br>GGAAATCTGCTGGCTCCGAGA	(Wei et al., 2012)
TSF-F<br>*TSF-R*	ATGCCTTTGGCAATGAGGTG<br>ACCGTTTGTCTTCCGAGTTG	
UPL7-F<br>*UPL7-R*	TTCAAATACTTGCAGCCAACCTT<br>CCCAAAGAGAGGTATCACAAGAGACT	(Czechowski et al., 2005)
XAL1-F<br>*XAL1-R*	CGAAAAGATAGAGGAAAACAAC<br>GGCATTGTTAGCGGATAGGA	

Czechowski, T., Stitt, M., Altmann, T., Udvardi, M. K., and Scheible, W. R. (2005). Genome-wide identification and testing of superior reference genes for transcript normalization in arabidopsis. *Plant Physiology* 139, 5-17. doi:10.1104/pp.105.063743.

Han, P., García-Ponce, B., Fonseca-Salazar, G., Alvarez-Buylla, E. R., and Yu, H. (2008). AGAMOUS-LIKE 17, a novel flowering promoter, acts in a FT-independent photoperiod pathway. *The Plant J* 55, 253-265. doi:10.1111/j.1365-313X.2008.03499.x.

Leivar P., Monte E., Al-Sady B., Carle C., Storer A., Alonso J. M., Ecker J.R., and Quail P.H. (2008). The Arabidopsis Phytochrome-Interacting Factor PIF7, Together with PIF3 and PIF4, Regulates Responses to Prolonged Red Light by Modulating phyB Levels. The Plant Cell, 20: 337-352. doi/10.1105/tpc.107.052142

Liu, C., Chen, H., Er, H. L., Soo, H. M., Kumar, P. P., Han, J.-H., et al. (2008). Direct interaction of AGL24 and SOC1 integrates flowering signals in Arabidopsis. *Development* 135, 1481-1491. doi:10.1242/dev.020255.

Tapia-López R., García-Ponce B., Dubrovsky J.G., Garay-Arroyo A., Pérez-Ruíz R.V., Kim S.H., Acevedo F., Pelaz S. and Alvarez-Buylla E.R. (2008). An *AGAMOUS*-related MADS-box gene, *XAL1* (*AGL12*), regulates root meristem cell proliferation and flowering transition in *Arabidopsis thaliana*. Plant Physiol. 146(3): 1182-1192. doi: 10.1104/pp.107.108647.

Wei, S., Gruber, M. Y., Yu, B., Gao, M. J., Khachatourians, G. G., Hegedus, D. D., et al. (2012). Arabidopsis mutant sk156 reveals complex regulation of SPL15 in a miR156-controlled gene network. *BMC Plant Biol* 12, 169. doi:10.1186/1471-2229-12- 169.

Table S2. Primers used in ChIP assays

Gene	Sequence
FD	
Fragment 1	F CCAAGGCCCTCTCTACTTGA
	R GATTGTGACATTGAACCTATTCT
Fragment 2	F CCATCTTGACTATCCAACGGT
	R TCCGTAGGTTGTCTCTAGAC
Fragment 3	F ACCCAATGTTTGGAAACATGTAA
	R TTATCGAAAATGCAAGCTCCTC
Fragment 4	F CATATAATAGTTTATGGAATTTCTC
	R ATGATGGGGTTGGGTCCAAG
SOC1	
Fragment 1	F CTCCCGATATAGATAAAAGATC
	R AGTAGCGACGTGTCTAAAGAG
Fragment 2	F TGAATTTTATCTGTTGGGATGG
	R ACACTCTCTCGTACCTATATG
Fragment 3	F GTGTTTGTGTCCACATTAAAAAC
	R GCTTGAAACCTCATCCTTTAC
Fragment 4	F CATTACCATAACTACAACGAGA
	R TTGATTCTCTGCGAAAGGAAG
LFY	
Fragment 1	F ATCCATTTTTCGCAAAGGAAAG
	R GGTTCACAAGAACGGAATCAC
Fragment 2	F TCTTCCCCTAACAATACTTCC
	R TTAACTGTATTGGCATGGACG
Fragment 3	F CCCAGCAAGACACATATCTTC
	R CTTTCTTTACTCTTTTGAGCAG
AP1	
Fragment 1	F TTGTTTTCTCTAAGGCTTATGC
	R GCTCAATTTGCTTAATCACTTC
Fragment 2	F CAACATAGCACATATTCAACTG
	R ACTGTGTAATGTATTGCGTCG
Fragment 3	F TAAAGATCCCGAGACTCAAAC
	R TACCCCTTCCCATTTTTGATC
Fragment 4	F GCCCTAATTATTCACTACTGC
	R TTCTAGGGTTCACTTCACTAC
